# The initiation of segmented buoyancy-driven melting during continental breakup

**DOI:** 10.1038/ncomms13110

**Published:** 2016-10-18

**Authors:** Ryan J. Gallacher, Derek Keir, Nicholas Harmon, Graham Stuart, Sylvie Leroy, James O. S. Hammond, J-Michael Kendall, Atalay Ayele, Berhe Goitom, Ghebrebrhan Ogubazghi, Abdulhakim Ahmed

**Affiliations:** 1 Ocean and Earth Science, National Oceanography Centre Southampton, University of Southampton, Southampton SO14 3ZH, UK; 2 Dipartimento di Scienze della Terra, Università degli Studi di Firenze, Florence 50121, Italy; 3 School of Earth and Environment, University of Leeds, Leeds LS2 9JT, UK; 4 Sorbonne Universités, UPMC - ISTEP - CNRS UMR7193, Paris 75252, France; 5 Department of Earth and Planetary Sciences, Birkbeck, University of London, London WC1E 7HX, UK; 6 School of Earth Sciences, University of Bristol, Bristol BS8 1RJ, UK; 7 Institute of Geophysics, Space and Astronomy, Addis Ababa University, Addis Ababa, Ethiopia; 8 Eritrea Institute of Technology, Asmara, Eritrea; 9 Seismological and Volcanological Observatory Center, Dhamar, Yemen

## Abstract

Melting of the mantle during continental breakup leads to magmatic intrusion and volcanism, yet our understanding of the location and dominant mechanisms of melt generation in rifting environments is impeded by a paucity of direct observations of mantle melting. It is unclear when during the rifting process the segmented nature of magma supply typical of seafloor spreading initiates. Here, we use Rayleigh-wave tomography to construct a high-resolution absolute three-dimensional shear-wave velocity model of the upper 250 km beneath the Afar triple junction, imaging the mantle response during progressive continental breakup. Our model suggests melt production is highest and melting depths deepest early during continental breakup. Elevated melt production during continental rifting is likely due to localized thinning and melt focusing when the rift is narrow. In addition, we interpret segmented zones of melt supply beneath the rift, suggesting that buoyancy-driven active upwelling of the mantle initiates early during continental rifting.

## Abstract (web summary)

Our understanding of melt production during early continental breakup remains poorly constrained. Using the Afar triple junction as an example, Gallacher *et al*. generate a 3D velocity model suggesting that melt production is highest during early continental breakup due to localised thinning of the crust.

## Graphical abstract

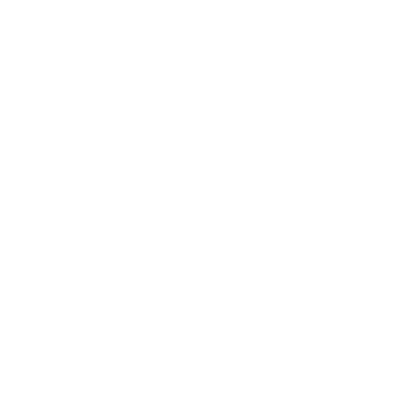

Explanations for melt production during rifting have largely been developed from models of mantle melting based on petrological and geochemical interpretations from rock samples and seismic velocities of igneous crustal intrusion at ancient rifted passive continental margins^1^,^2^,^3^,^4^. Due to the lack of direct observations, however, the relative importance of mantle potential temperature^5^, mantle composition^2^, melt focusing along the lithosphere–asthenosphere boundary (LAB)^6^ or prior rift history^1^ in determining the locus and volume of melt production are debated. In addition, the importance of buoyancy-driven active upwelling in controlling the three-dimensional (3D) geometry of melt production during rifting remains unclear.

Rifting in the Horn of Africa provides a unique opportunity to directly constrain melting processes since the region exposes several stages of rift sector development from continental rifting in the Main Ethiopian Rift (MER) to seafloor spreading in the Red Sea and Gulf of Aden (GOA). Rifting in the Red Sea and GOA started at ∼30 Ma, whereas in the central and northern MER (CMER and NMER, respectively) rifting started 11–18 Ma (ref. 7). Crustal extension during Oligocene-Miocene occurred primarily on border faults. Subsequently, strain shifted to localized dyking within ∼70-km-long, ∼20-km-wide Quaternary-Recent volcanic segments^7^. Extension and associated lithospheric thinning has facilitated upwelling and melting of the mantle creating a magma-rich extensional setting^8^.

Here, we use an array-based teleseismic surface wave tomography method, on a dense network of 290 broadband seismographs (Fig. 1) spanning the continent-ocean transition to create the first high-resolution 3D shear velocity model for the region. This can resolve changes in mantle structure caused by extension-related melt production. This method improves on previous relative arrival-time tomography in the region^9^,^10^,^11^ by providing greater vertical resolution in the expected melt zone (∼40–150 km) and absolute shear wave velocity variations from which causes of seismic heterogeneity can be more easily explained. Our model also builds on previous low-resolution regional and global surface wave studies imaging the African superplume^12^. Additional independent constraints on melting conditions and depth provided by recent petrological studies aids the interpretation of these results^13^,^14^,^15^,^16^.

## Results

### Regional phase velocity results

Using teleseismic Rayleigh waves we obtain an average phase velocity dispersion curve for periods of 20–125 s (Fig. 2a) and two-dimensional (2D) phase velocity maps at each period including terms for azimuthal anisotropy. We organize our anisotropy inversions to correspond to the primary tectonic environments (MER, Afar, Nubian and Somalian Plateaux, Yemen, Red Sea and GOA; Supplementary Fig. 1). We obtain an average phase velocity dispersion curve for each of these tectonic environments (Fig. 2a). Regionalized phase velocity curves for the areas indicated in Supplementary Fig. 1 show that overall the entire study region is anomalously slow for a continental setting, with the average phase velocities at all periods being <4.0 km s^−1^ (Fig. 2a). Relative to the average dispersion and all other dispersion curve for the entire region, the MER has lowest phase velocities at all periods. These suggest that the source of low velocities at the MER is distributed throughout a larger range of depths compared with the other regions given the depth sensitivity of Rayleigh waves in this period range (Fig. 2b). Afar and the GOA have phase velocities that are similar to the regional 1-D average, likely owing to the distribution of the data used in the study. The Plateaux and Yemen are faster than the average dispersion for the entire region from 20–60 s periods by up to 0.05 km s^−1^, indicating that the uppermost mantle is faster in these regions given the depth sensitivity at these periods (Fig. 2b). The Red Sea phase velocities are higher at 30–60 s periods by up to 0.04 km s^−1^.

### Shear velocity results

To determine the best-fit one-dimensional (1D) shear velocity model we invert the average phase velocity dispersion curve using depth sensitivity kernels (Fig. 2). The output shear velocities are up to 11% lower than ak135 at depths of 40–150 km, indicating that the mantle shear velocities in the study area are significantly lower than the global average (Fig. 2c). We then perform the same 1D shear velocity inversion for each point in the 2D phase velocity maps across all periods to compute the 3D shear velocity model. This enables us to constrain the spatial extent and 3D character of variations in shear velocities in the likely asthenospheric melt zone. Figure 3 shows the 40–132 km average depth slice, which gives a fully resolved minimum structure view of our model. Analysis of formal resolution and Backus–Gilbert resolution kernels (Supplementary Fig. 2) indicates that velocity anomalies are smoothed by up to ±50 km in this depth range.

At mantle depths (>40 km), shear wave velocities of <4.00 km s^−1^ underlie the rift, which are lower than those observed beneath the adjacent flanks (>4.05 km s^−1^; Fig. 3). These lower wave-speeds are not homogeneous; instead shear wave velocities of <3.90±0.06 km s^−1^ are segmented beneath the rift with each lobe typically 200–300 km long and a regular spacing between lobes of 100–150 km (Fig. 3a). These lowest velocities are specifically focused in four regions, the CMER, NMER, Afar and the GOA. Checkerboard tests of the 2D phase velocity maps at 40 and 71 s (Supplementary Fig. 3) show that anomalies with length scales of ∼150 km are well-resolved, allowing interpretation of these features. The checkerboard tests also suggest that shear velocity anomalies of this size may be underestimated by up to ±0.05 km s^−1^. The maximum depth extent of the anomalies varies along the rift from 120±55 km beneath the less mature CMER to 80±45 km beneath the more mature Afar. In addition, the mantle low-velocity anomalies do not directly underlie either the surface volcanic segments or the lowest crustal velocities. The mantle beneath the border faults, rift flanks and surrounding plateaux away from the CMER anomaly is characterized by higher-velocities (>4.1 km s^−1^).

Azimuthal anisotropy obtained from the 2D phase velocity maps is presented, in Fig. 2d, as the percentage variation from the background shear velocity by node at each period. For periods <50 s rift parallel fast directions are observed for the MER and GOA with up to 10% and ∼1% peak-to-peak anisotropy, respectively. The Red Sea has strong anisotropy, up to 10% peak-to-peak, with azimuths mostly NNE-SSW. Afar and the surrounding plateaux have negligible anisotropy at short periods agreeing with interpretations from previous SKS studies regarding shallow anisotropy as resulting from crustal melt alignment^17^,^18^. The magnitude of anisotropy beneath Yemen is up to 5% peak-to-peak striking mostly NE-SW (Fig. 2d). For all regions, at periods >50 s, almost all anisotropy is >5% peak-to-peak with azimuths between 320° and 30°.

### Previous studies

These results while broadly consistent with previous tomographic studies of the region, summarized below, provide laterally and vertically well-resolved absolute shear wave velocities, which allows us to infer values for mantle potential temperature and attenuation. This cannot be done using the relative velocities imaged by previous tomographic studies. Thus our model is the first in the region that can be used to estimate percentage melt in the mantle beneath Afar and the NMER. In addition, our model solves for mantle azimuthal anisotropy, which other regional models do not account for, allowing us to potentially distinguish between anisotropic lowering of shear velocities and the effect of partial melt.

Bastow *et al*.^19^ perform relative arrival-time body wave tomography of the MER and find shear velocities 4% lower than the background mean focused beneath the CMER in the same location as observed in this study. Their low shear velocities extend deeper into the upper mantle than we observe, possibly due to the vertical ray path smearing indicated by their resolution tests. The focused low shear velocities are also observed by Bastow *et al*.^9^ who use a larger network, sampling more of the rift flanks. Bastow *et al*.^9^ has improved depth resolution compared with the previous tomography and the focused low-velocity anomaly does not extend to depths >250 km. Hammond *et al*.^10^ build on the model of Bastow *et al*.^9^ using data from Afar and Eritrea. They also find shear velocities 2% lower than the background mean directly beneath the rift extending from the MER into Afar^10^.

Kendall *et al*.^20^ constrain anisotropy in the mantle beneath the MER using SKS splitting. They find strong anisotropy, >2 s of splitting, with fast polarization directions orientated NE-SW within the rift^20^. Fast polarization directions outside the rift are also orientated NE-SW with reduced splitting magnitudes. The direction and magnitude of anisotropy for our short period results, <50 s, agree well with this result and support the interpretation of aligned melt contributing to anisotropy within the rift. Hammond *et al*.^18^ also use SKS splitting to constrain anisotropy throughout Ethiopia. They invert for multiple layers of anisotropy and interpret that vertically aligned melt is present in the uppermost mantle beneath the MER, with aligned olivine from mantle flow below^18^. In Afar they show that in general azimuthal anisotropy in the shallow mantle is low and that splitting is caused by olivine alignment from mantle flow. They interpret this as suggesting that melt has little preferential orientation or that melts are predominantly aligned in horizontal structures. Exceptions to this are at border faults and close to volcanic segments, where shear-derived segregation of melt^6^ or alignment beneath the rift axis^18^,^21^ generate vertically aligned melt. Given surface-waves lower lateral resolution, and thus relative insensitivity to localized strong anisotropy, our anisotropic models support this conclusion, showing lower azimuthal anisotropy beneath Afar.

## Discussion

The CMER is characterized by especially low velocities (3.85–4.00 km s^−1^) in the mantle and low crustal velocities of <3.60±0.10 km s^−1^, which may be artificially low due to anelasticity not accounted for in our shear velocity inversion. We estimate the maximum velocity increase from attenuation by inverting 1D profiles while accounting for attenuation^22^, with a shear Q factor of 60 (ref. 23) at periods of 20–125 s. We find that high attenuation, Q factor ∼60, can decrease shear velocities at melting depths by up to ∼0.20 km s^−1^. Thus, in the CMER high attenuation results in observed shear velocities that are ∼0.20 km s^−1^ lower than in the case with no attenuation. If the effect of attenuation, 0.20 km s^−1^, is accounted for in our observed shear velocities the range of the lowest velocities increases to 4.05–4.20 km s^−1^, with shear velocities beneath the border faults, rift flanks and surrounding plateaux of 4.30–4.40 km s^−1^, in better agreement with expected velocities of mantle peridotites^24^. As both of these regions velocities remain lower than ak135 (Fig. 2c) and low Q may be required, an increase in mantle potential temperature or partial melt is needed.

A mantle potential temperature of 1,450 °C has been estimated for the region from modelling REE compositions of recent basaltic lava flows^13^,^15^,^16^. This can account for the difference between the un-rifted plateaux and ak135 and has been suggested as being due to the presence of a broad mantle plume encompassing the whole region^25^. To explain the observed shear velocities at the CMER we use a Burgers model relating viscoelasticity and temperature^26^ with a rifting rate of ∼6.0 mm per year^27^ and a half space cooling model to generate the geotherm for the MER to determine the mantle potential temperature needed to match our observation. This requires iteratively varying the mantle potential temperature input to the Burgers model until the predicted shear velocities in the melt zone match with those observed in our model. We estimate a mantle potential temperature of ∼1,700 °C would be required to explain the attenuation corrected low velocities (0.2 km s^−1^ added relative to the regional average shear velocity in Fig. 2c) with temperature anomalies alone; 250 °C greater than petrological estimates^14^. A mantle potential temperature of this value would lead to significant amounts of partial melt within the depth range imaged by our study. In addition a value for attenuation of 60 also suggests partial melt is present in the mantle. We therefore appeal to the presence of partial melt, which is an effective way of significantly lowering shear velocities, to account for the discrepancy in shear velocity between the petrologically determined mantle potential temperature and that estimated from our results. Given that the mantle potential temperature estimated from our results for the surrounding plateaux is ∼1,450 °C we propose that the lower shear velocities in the rift compared with the surrounding plateaux are due to partial melt. We use the experimental relationship of 1% partial melt lowering shear velocities by 7.9% (ref. 28) to estimate ∼0.6% partial melt for an isotropic mantle. Additional constraints for the amount of partial melt in the CMER come from seismic anisotropy. Both our results and previous studies indicate strong anisotropy in the CMER mantle, interpreted as vertically aligned melt inclusions with low aspect ratios^17^,^18^. Analytical models of the effect of melt pocket geometry on wave-speeds shows that vertically aligned melt inclusions can cause more extreme velocity reductions (for example, aspect ratios of ∼0.03 provide 11% shear velocity reduction for 1% partial melt^29^) suggesting that even smaller melt fractions, <0.5% partial melt, can explain our observed anomalies beneath the CMER.

The Afar and GOA mantle anomalies (3.90–4.00±0.06 km s^−1^) are also difficult to explain with elevated temperatures alone, even after accounting for the 0.2 km s^−1^ velocity increase correcting for attenuation^22^,^23^. Using the same method as for the CMER, but with a rifting rate of 16 mm per year (ref. 30) and a half space cooling model to generate a geotherm applicable for Afar, an elevated mantle temperature of 1,650 °C would be required to explain seismic velocities^26^. In addition the temperature anomaly expected to result from lateral advection and conduction of heat in response to lithospheric thinning is also insufficient to explain our low velocities^13^. Instead, the experimental relationship giving 7.9% shear velocity reduction^28^ yields an interpretation of 0.3% melt in the melt zone. The magnitude of seismic anisotropy is relatively low in Afar, meaning our estimates of per cent partial melt are not significantly altered after accounting for azimuthal anisotropy. Radial anisotropy due to horizontally aligned melt could reduce the amount of melt required to match the low velocities.

The depth range of interpreted melt zones identified in our model compare well with petrological constraints, where we interpret melt generation is responsible for low shear velocities at depths >∼75 km and melt retention is responsible for shallower low shear velocities. For example, the 120±55 km depth to the base of the interpreted melting region, beneath the CMER is broadly consistent with the petrologically constrained melting depths of 53–88 km (ref. 14). Beneath Afar the petrologically defined melting depths are up to 81 km (ref. 13), in line with the maximum depth of interpreted melting at 80±45 km. Our model for Afar is also consistent with the 75-km depth for the onset of melting constrained from modelling of S-p receiver functions^31^.

The relatively shallow maximum depth of 120±55 km for interpreted melting combined with the position within the rift strongly suggest that decompression melting in response to extension is the dominant source of partial melt. The spatial offset of the melting from zones of crustal intrusion and the interpreted segmented nature of upwelling cannot, however, be explained by decompression melting occurring by passive upwelling. The 3D diapiric nature of partial melt, which is interpreted throughout the region, is more indicative of active upwelling^32^. Other possible explanations for the diapiric and segmented nature of partial melt include geochemical or temperature variations associated with the impact of one or more plumes. Pik *et al*.^33^ demonstrated that 3He/4He ratios (Ra) of volcanic rocks within Afar and the MER span crustal, normal mid-ocean ridge basalt and elevated values. These elevated values are located in both the MER and Afar and suggest that material from the lower mantle has been mixed into the melt zone in the upper mantle. This has previously been used as evidence of the presence of a mantle plume and provides support for a hypothesis of multiple small plumes within the rift potentially explaining the observed segmentation in our model. However despite the presence of elevated Ra values petrological studies, including Pik *et al*.^33^, have shown that there is no consistent variation in mantle potential temperature or geochemical composition within the region^15^,^33^. While volcanism within the rift exhibits variations in the inferred melting column depth^34^ and mantle source regions^35^, these variations can be observed within individual volcanic segments. Thus while these results suggest that the mantle may be heterogeneous, the heterogeneity is on a significantly smaller scale than the mantle segmentation interpreted in this study. Although we cannot exclude the possibility that there are multiple small plumes within the rift we suggest, given previous observations of short length scale geochemical variations, that it is more likely that elevated Ra values are reworked material from the initial plume impact ∼30 Ma, as has been suggested from geodynamical studies of plume/mantle entrainment^36^. In addition the shallow maximum depth, ∼150 km, of the observed anomalies is contrary to what would be expected if the observations were due to the impact of a plume assuming that the plume conduit was continuous. Given the interpretation of volumes of melt comparable to oceanic spreading centres^37^ present in the asthenosphere, buoyancy due to enhanced melt retention is the most likely cause of active upwelling resulting in enhanced decompression melting^31^.

The maximum depth of low wave-speeds is greatest beneath the CMER, the youngest and least mature rift in our system. This observation is consistent with both a deeper onset and increased amount of partial melting. Shallower melt and reduced volumes of partial melting are interpreted to be beneath Afar, an older and more mature rift. To explain this reduction in melt volume between the CMER and Afar requires a further process beyond active upwelling. The younger age of the CMER may result in greater melt production than for Afar at similar mantle temperatures. Alternatively, a decrease in mantle temperature as rifting proceeds could also lead to reduced melt production^3^. Equally plausible is that the lithosphere at this section of the CMER was thinned by prior rifting before the onset of extension and thermal anomaly in the Oligocene, which numerical models predict will enhance melt production^1^.

The interpretation of off-axis melt production suggests that lateral melt migration occurs within the mantle lithosphere or at the base of the crust before intrusion^6^. In the relatively narrow CMER, 3D melt focusing at steep LAB topography and shear-derived segregation of melt due to high strain rates combined with along-rift thinning of lithosphere into Afar may enhance melting^6^. This process relies on the presence of steep gradients on the LAB near the locus of rifting and is therefore most effective in a narrow rift, melt focusing would likely be subdued in the more developed Afar rift^18^. We cannot preclude the possibility, however, that we are imaging transitory melt migration, where the upwelling melt volume is abnormally high before eruption.

Our results support decompression melting controlled by buoyancy-driven active upwelling being dominant during continental rifting and incipient seafloor spreading. This is consistent with interpretations of higher melt production during the transition from continental to oceanic rifting from observations of increased oceanic crustal thickness at many oceanic rifts^38^,^39^. In addition, increased active upwelling has been proposed as the source of the interpreted higher melt production at oceanic rifts^40^, in agreement with the findings of this study. Off-axis zones of segmented melt production have also been observed at more developed oceanic rifts, including the Gulf of California and the Red Sea^41^,^42^,^43^. These segmented anomalies are explained by a combination of buoyancy-driven active upwelling and melt migration^41^. The similarity between the observed segmentation in our study and segmentation at more developed oceanic rifts, with spacing between segments of 75–150 km, suggests a similar origin for discrete zones of enhanced mantle melting. In addition recent publications support the view of future progression of the rifting in Afar towards seafloor spreading^44^,^45^. Thus the early onset of buoyancy-driven active upwelling during continental rifting and continuation until seafloor spreading implies that the segmentation of melt zones in the mantle at oceanic rifts is initially controlled by the locus of upwelling during continental rifting. We observe larger degrees of melting than at fully developed mid-ocean ridges and interpret the increased melt volumes as resulting from moderately increased mantle temperatures and on-going lithospheric thinning producing significant lithospheric topography compared with steady-state melt production beneath new igneous crust during seafloor spreading.

## Methods

### Station and event data

**Figure 1:**
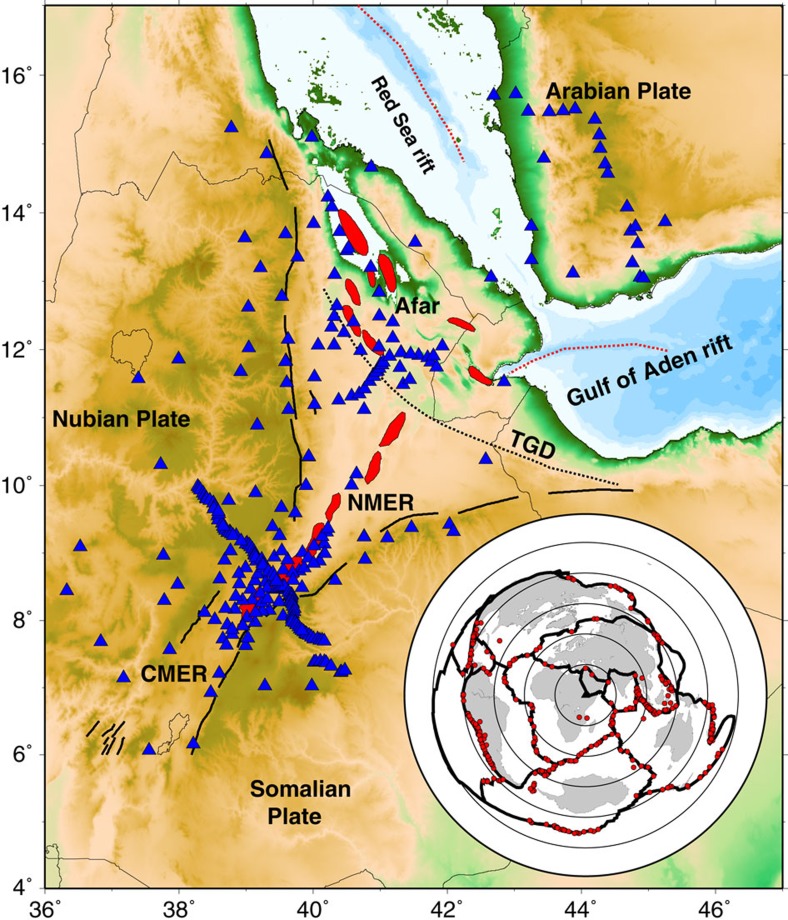
Network configuration and magmatic segments in the Horn of Africa. The blue triangles are broadband seismic stations. The Tendaho-Goba'ad Discontinuity (TGD), shown as a black dotted line, marks the boundary between the NMER and Afar. Red dotted lines are the rift axes of the submarine Red Sea rift and the GOA. The broken black lines show the border faults for the MER and Afar. The magmatic segments for Afar, CMER and NMER are shown shaded in red. In the inset figure, red dots are the locations of the 569 earthquakes used to create the 3D model showing the good back azimuthal coverage of the earthquakes.

**Figure 2:**
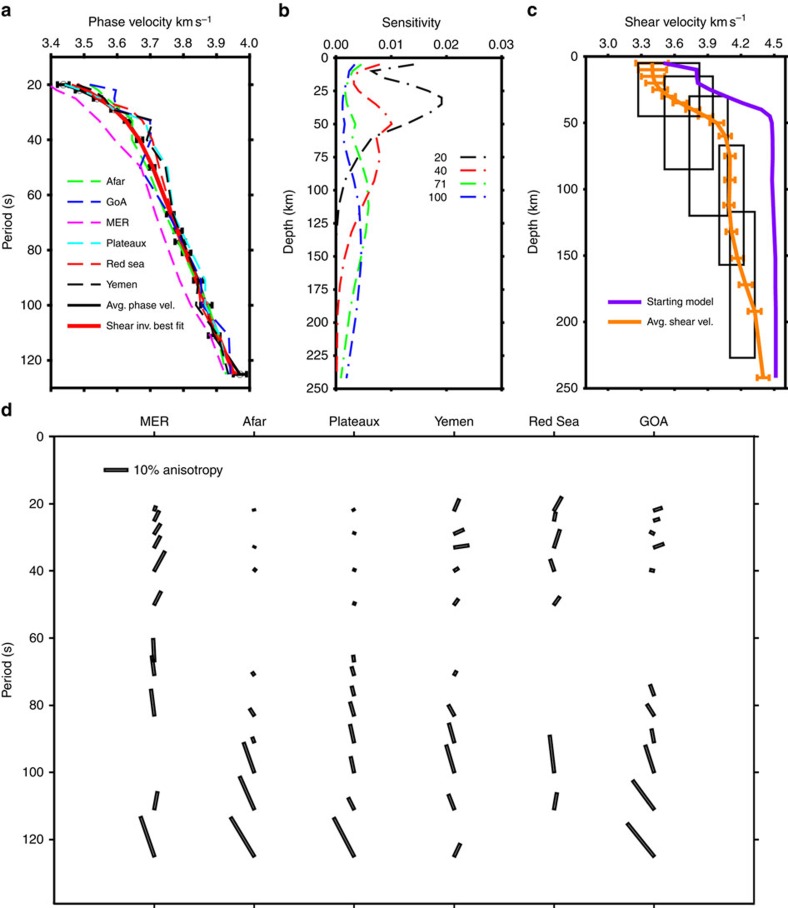
1D shear velocity structure and phase velocity dispersion curves with anisotropy profiles. (**a**) The predicted phase velocity dispersion curve (red) obtained from the average 1D shear velocity profile for the region fits well with the average phase velocity dispersion curve (black) for the whole region. Black horizontal bars show 3 × s.e. Regional phase velocity dispersion curves (dashed) are determined using a subset of the nodes used for the whole region. (**b**) The sensitivity kernels calculated from DISPER80 (ref. 58) are shown for representative periods between 20 and 100 s. (**c**) The average regional 1D shear velocity model (orange) is up to 11% lower than the starting model (purple), which combines CRUST 1.0 (0–40 km) and ak135 (40–250 km), at depths >50 km. Black rectangles show the average error in shear velocity over well-resolved depth ranges. Errors are obtained from a Monte-Carlo estimate (100,000 random perturbations from our best-fit model) showing the range of possible solutions to the inversion that fit the dispersion curve within error. Resolution at 25, 50, 75, 112 and 167 km is shown as vertical black lines. (**d**) Magnitude and azimuth (black rectangles) of anisotropy for our well-resolved periods is shown. The region was divided into six separate zones each solved separately for anisotropy.

**Figure 3:**
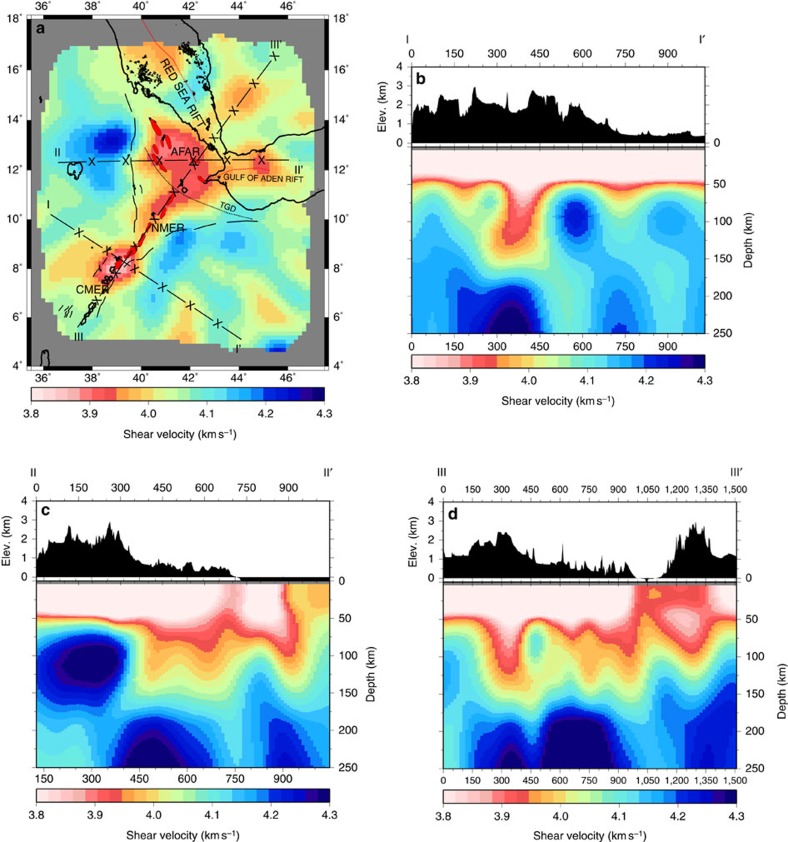
Depth-averaged 3D model slice and transects. (**a**) The 40–132 km averaged depth slice shows that the GOA and Afar form a continuous low velocity (<4.00 km s^−1^) region. Higher velocities (>4.00 km s^−1^) separate low velocities between the NMER (3.90–4.00 km s^−1^) and a focused low velocity (3.80–3.95 km s^−1^) along the CMER. Transects are labelled I-I', II-II' and III-III'. X's mark intervals of 150 km on the transects. The Tendaho-Goba'ad Discontinuity (TGD), shown as a black dotted line, marks the boundary between the NMER and Afar. Red dotted lines are the rift axes of the submarine Red Sea rift and the GOA. The broken black lines show the border faults for the MER and Afar. The magmatic segments for Afar, CMER and NMER are shown shaded in red. (**b**) The I-I' transect shows a focused low velocity (3.80–4.00 km s^−1^) beneath the CMER. (**c**) The II-II' transect shows low velocities beneath Afar (3.80–4.00 km s^−1^) and beneath the GOA (3.80–4.00 km s^−1^). (**d**) The III-III' transect shows an ∼200 km long low velocity (3.80–4.00 km s^−1^) beneath the CMER and an ∼300 km long low velocity (3.90–4.00 km s^−1^) beneath the NMER and Afar. The spacing between the low velocities is 150 km. Crustal velocities <3.8 km s^−1^ are saturated at 3.8 km s^−1^.

We used Rayleigh-wave vertical component records for earthquakes recorded at 290 broadband stations from 12 temporary seismic deployments during 1999–2013, and 6 permanent stations; AAUS, ANKE, ATD, DAMY, FURI, RAYN (Supplementary Fig. 4). The temporary seismic deployments are; the Reseau Large Bande Mobile Horn of Africa experiment (RLBM: June 1999–December 2002)^46^, Ethiopia Kenya Broadband Seismic Experiment (Ethiopia/Kenya: January 2000–December 2002)^25^, Ethiopia Afar Geoscientific Lithospheric Experiment (EAGLE: October 2001–February 2003)^9^, Boina Broadband Network (Boina: October 2005–October 2007)^21^, SEARIFT array (SEARIFT: March 2007–October 2009), UK-US Afar Consortium Experiment (Afar Consortium: October 2007–October 2009)^47^,^48^, Danakil Depression array (Afar Depression: October 2009–February 2013), Eritrea Seismic Project (Eritrea: June 2011–October 2012)^10^ and Young Conjugate Margins Laboratory in the GOA (YOCMAL: March 2009–February 2010)^49^. In total, 569 teleseismic events with Ms >5.5 and epicentral distances of 25–150° were used (Fig. 1). The records had the mean, trend and instrument response removed.

### Node structure and anisotropy subdivision

The node structure for the tomography is seen in Supplementary Fig. 1 and has 25 × 29 nodes covering the region of interest. The node spacing is 0.5° × 0.5° with the outermost two rows and columns spaced at 1° to absorb velocity heterogeneities outside the region of interest. The variation of velocities allowed at the outside edges is increased by a factor of 10 to allow for this. The nodes are subdivided into seven regions to solve for anisotropy in the 2D phase velocity maps. The regions are labelled as; MER, Afar, Nubian and Somalian Plateau (Plateaux), Yemen, Red Sea, GoA and external nodes. Supplementary Fig. 5 shows ray path maps for events used in this study for 20, 40, 71 and 100 s periods.

### 1D phase velocity inversion

To solve for the phase velocity we invert amplitude and phase information extracted from the individual seismograms by minimizing the misfit in both the real and the imaginary components of the bandpass-filtered seismograms. We use an array method which accounts for distortions in the incoming wavefield using a two-plane wave approximation^50^ and accounts for first order scattering using 2D Born approximation sensitivity kernels for amplitude and phase^51^,^52^. The inversion is completed in two stages, with the first stage utilizing a simulated annealing method^53^ and the second stage utilizing a standard linearized inversion technique^54^. The inversion simultaneously solves for the phase velocity, azimuthal anisotropy and wave parameters for each event. The first stage is necessary due to the periodic non-linearity of the problem, in addition to the solution being ambiguous where the two-plane waves have similar azimuths. By performing the simulated annealing inversion first we ensure a global minimum is found in the second stage.

We determine the average dispersion curve for the region using a 1D version of the two-plane wave inversion method, which solves for the 6 wave parameters for each event and a single phase velocity for the region. We try a range of starting phase velocities for the model ranging from 3.00–4.00 km s^−1^, typically the models converge on a single phase velocity for each period. Thus we use an average of the output from these starting velocities as our starting model. After an initial set of inversions for the period range of interest, we quality control the data and assess the quality of fit, before a final set of inversions for the final 1D dispersion curve. The quality control consists of identifying events that cannot be fit with a plane wave model, either due to incoherent propagation across the array or complications in the sources.

### 2D phase velocity inversion

For the 2D phase velocity maps we start with a uniform velocity at each point across the map where the value is taken from our 1D dispersion curve for each period of interest. Again any event >0.05 out of phase is removed and the inversion is repeated using the output phase velocity maps as input for the following iteration. The culling of this data is necessary as it removes waveforms with complicated source radiation patterns and other effects not accounted for in the inversion. At short periods, a single average phase velocity may not adequately capture rapid variations in crustal structure across the region, especially given the transitions from continental to oceanic crust. To account for this, we repeated the 2D phase velocity inversions using the predicted phase velocity structure from the CRUST 1.0 model (ref. 55) and a homogeneous mantle velocity structure derived from our best-fit 1D shear velocity model as our starting model. The 2D phase velocity maps and azimuthal anisotropy for periods 20, 40, 71 and 100 s are shown in Supplementary Fig. 6, where azimuthal anisotropy was solved simultaneously with 2D phase velocities.

### Shear velocity inversion

For the shear velocity inversion we used the Occam inversion^56^, which linearizes the nonlinear forward problem for a starting model, in our case a combination of CRUST 1.0 from 0–40 km depth and ak135 (ref. 57) from 40–250 km. This procedure has been designed to produce a smooth output model for shear velocity, which minimizes the potential for spurious features to be overinterpreted. The smoothness is achieved by parameterising the model for a roughness factor taken as the integrated square of the first derivative with respect to depth for the shear velocity model. The linearized problem is then solved for the desired model, rather than for a model correction, where the roughness factor is smallest while still fitting the data. This method is combined with the solutions for the Jacobian matrixes found by DISPER80 (ref. 58) to output a 1D shear velocity profile at a given point accounting for all periods. The 3D shear velocity model is generated by performing the above procedure for each individual point across all of the 2D phase velocity maps. The model is shown as depth-averaged slices in Supplementary Fig. 7 as this represents a minimum structure view of the model. The average 1D shear velocity profiles for each region are shown in Supplementary Fig. 8 and Supplementary Table 1 contains the values and errors for the average 1D shear velocity profile.

### Burgers model input

The age of the lithosphere for which shear velocity is calculated is defined as a distance from the axis of a rift. This distance is determined by multiplying the age of the rift by the average rifting rate. For Afar the rifting rate is 1.6 cm per year (ref. 30) and the rift started ∼30 Ma (ref. 7), giving a distance of 480 km. For the MER the rifting rate is 0.6 cm per year (ref. 27) and the rift started ∼11 Ma (ref. 7), giving a distance of 66 km. The other input is mantle potential temperature, which determines the geotherm. This is varied iteratively until the output shear velocity matches with the observed shear velocities.

### Formal resolution

We assess the lateral resolution of our model using a measure based on the formal resolution matrix of the linearized inversion for the 2D phase velocity maps. We present the values of the diagonal resolution matrix, which provides an indication of the independence of each velocity node (0 indicating not resolved, 1 meaning perfectly resolved, with numbers in between indicating fractional interdependence with adjacent parameters). This diagonal resolution plot is shown for the 20-, 40-, 71- and 100-s 2D phase velocity maps (Supplementary Fig. 9). The maps show independence values of 0.3–0.45, 0.3–0.6, 0.3–0.4 and ∼0.3 respectively within the interpreted regions. This means that a minimum of approximately three nodes is required to have a well-resolved model feature. As the node spacing is 50 km we can thus interpret features of 150 km or greater.

### Checkerboard tests

To further test the resolution of our model we perform ‘checkerboard' tests where we create synthetic 2D phase velocity maps for the 40- and 71-s period with the velocity blocks varying between 3.9 and 4.1 km s^−1^ at a length scale of 150 km. Supplementary Fig. 3 shows checkerboard outputs for 40- and 71-s with 150 km input checkers. It can be seen that the checkerboard structure is retrieved for both periods with slight underestimation of the anomalies (∼0.05 km s^−1^), which matches well with the result from formal resolution. To test the ability of the model to retrieve a continuous low-velocity region throughout the MER and Afar we create a synthetic 2D phase velocity map for the 40-s period with the low-velocity set to 3.9 km s^−1^ and the rest of the region set to 4.0 km s^−1^.

Supplementary Fig. 10 shows a synthetic 2D phase velocity map and the subsequently recovered velocity structure. This shows that segmentation of the model is a real feature and is not due to the inversion process. To check for potential smearing of anomalies along the NMER we create synthetic 2D phase velocity maps for a focused low velocity of 3.7 km s^−1^ at the NMER and South of the NMER. Supplementary Fig. 11 shows both synthetic 2D phase velocity maps and the subsequently recovered velocity structure at 40-s period. This shows that there is little smearing along the NMER suggesting that the low velocities beneath the NMER are well-resolved.

To test the vertical resolution we present Backus–Gilbert resolving kernels^59^ for a range of depths in our model (Supplementary Fig. 2). These kernels show the recovery of a delta function at the target depth and are based on the formal resolution matrix. This shows that shear velocities can be smeared over±25 km at 20 km depth, ±45 km at 75 km depth and±55 km at 112 km depth. Where these values are obtained for the well-resolved part of the kernel, which is defined as 0.025 for 20 km, 0.075 for 75 km and 0.1 for 112 km.

### Data availability

The authors declare that the data supporting the findings of this study are available within the article and its Supplementary Information Files.

## Additional information

**How to cite this article:** Gallacher, R. J. *et al*. The initiation of segmented buoyancy-driven melting during continental breakup. *Nat. Commun.* **7,** 13110 doi: 10.1038/ncomms13110 (2016).

## Supplementary Material

Supplementary Information Supplementary Figures 1-11 and Supplementary Table 1
