# Supplementary material for: The initiation of segmented buoyancy-driven melting during continental breakup
Source: Nat Commun. 2016 Oct 18;7:13110. doi: 10.1038/ncomms13110 (PMC5071842; doi:10.1038/ncomms13110)
Supplement: Supplementary Information — Supplementary Figures 1-11 and Supplementary Table 1 [file ncomms13110-s1.pdf]

Supplementary Figures

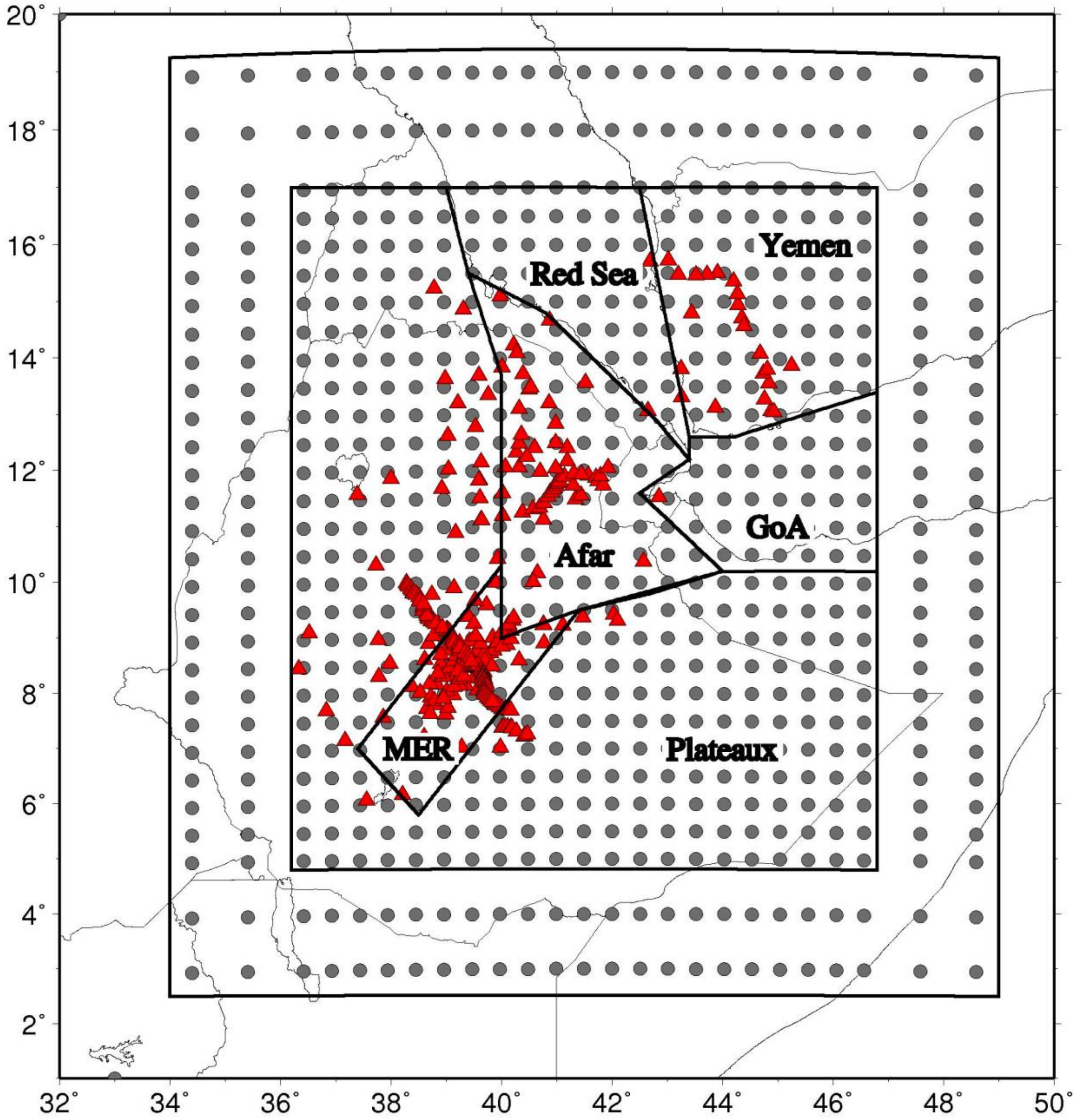

Supplementary Figure 1) Map showing node distribution throughout the region. Grey circles show nodes with stations used overlain (red). Subdivisions for solving anisotropy are named and shown in black. MER is Main Ethiopian Rift and GoA is Gulf of Aden.

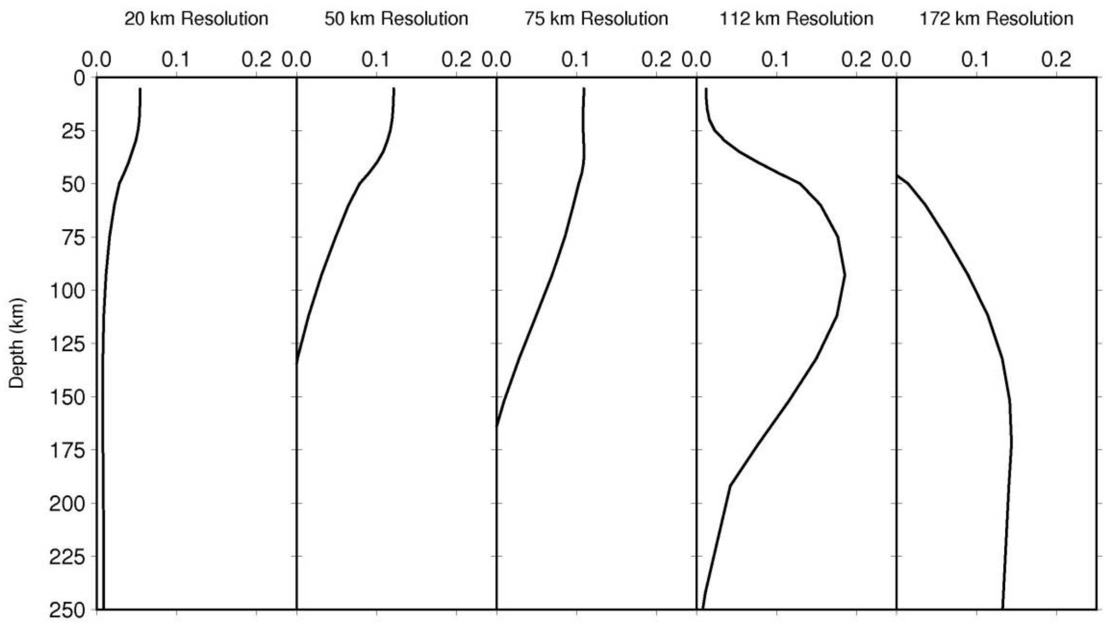

Supplementary Figure 2) Backus-Gilbert resolution kernels. Backus-Gilbert resolution kernels for depths of 20 km, 50 km, 75 km, 112 km and 172 km. Smearing ranges from ± 25 at 20 km depth to ± 55 km at 112 km depth.

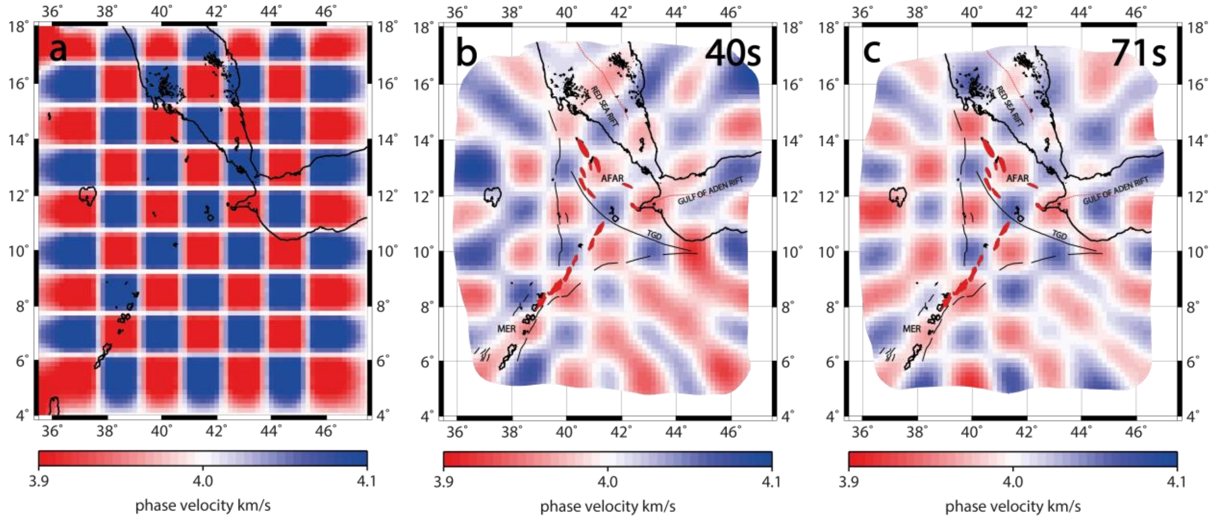

Supplementary Figure 3) Checkerboard tests. Checkerboard tests of 150 km scale checkerboard input **(a)** for the 40-second period 2-dimensional phase velocity map **(b)** and the 71-second period 2-dimensional phase velocity map **(c).**

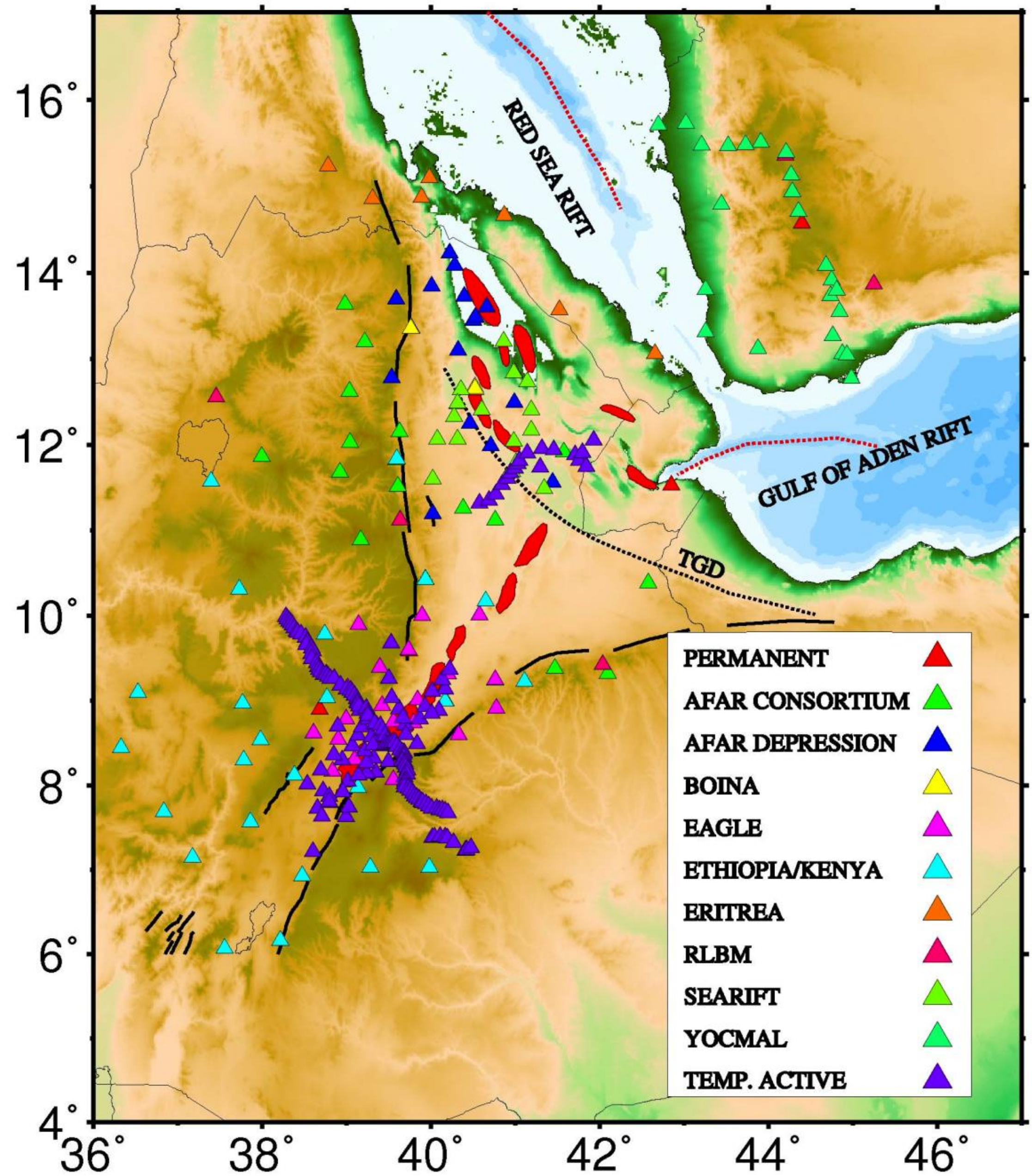

Supplementary Figure 4) Colour-coded network map. Colour-coded network map showing the different temporary deployments combined in this study. The Tendaho-Goba'ad Discontinuity (TGD), shown as a black dotted line, marks the boundary between the Northern Main Ethiopian Rift (NMER) and Afar. Red dotted lines are the rift axes of the submarine Red Sea rift and the Gulf of Aden (GOA). The broken black lines show the border faults for the Main Ethiopian Rift (MER) and Afar. The magmatic segments for Afar, Central Main Ethiopian Rift (CMER) and NMER are shown shaded in red.

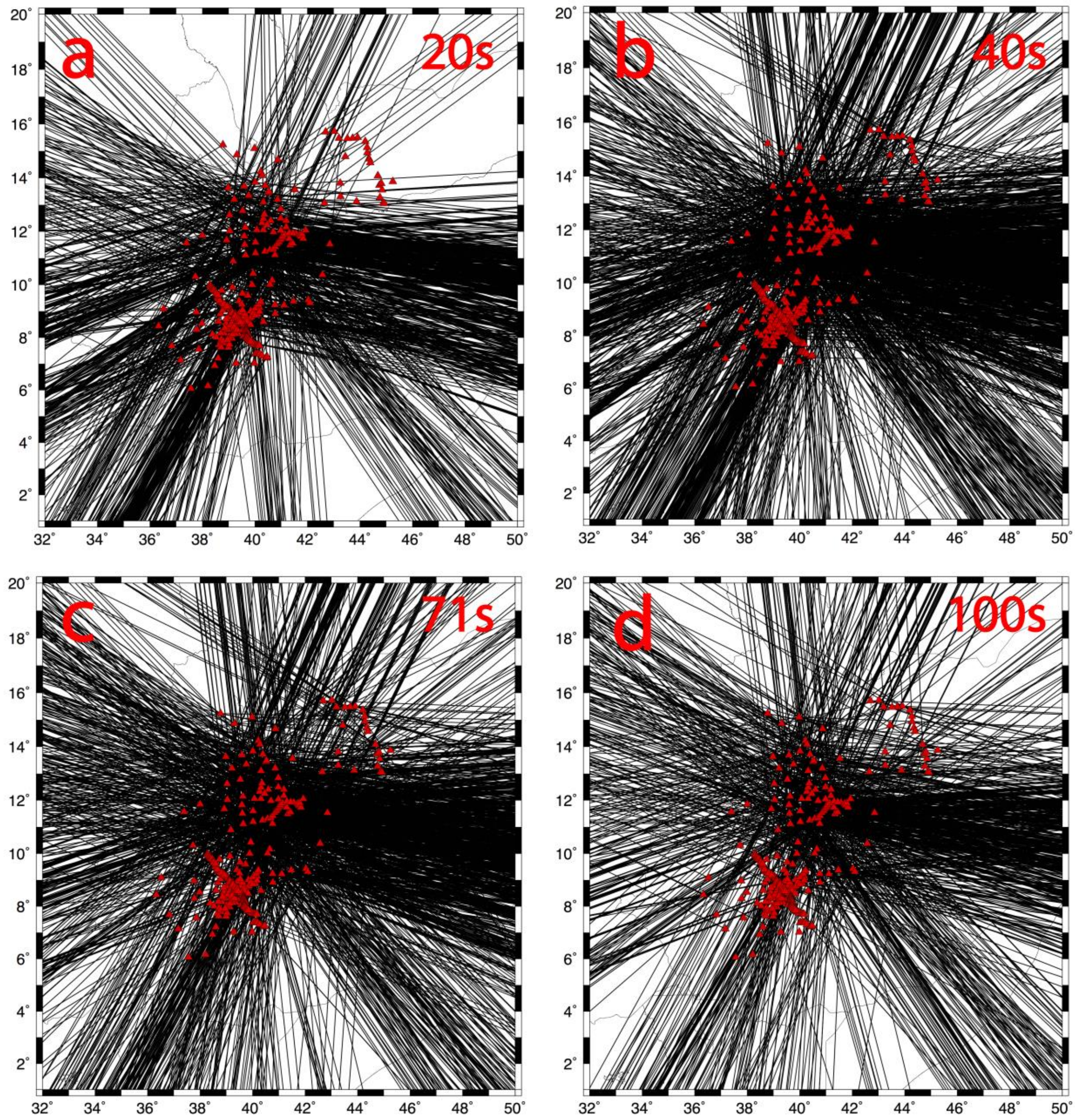

Supplementary Figure 5) Raypath maps. Raypath maps for 20s, 40s, 71s and 100s.

Coverage of crossing raypaths is good within the centre of the study region.

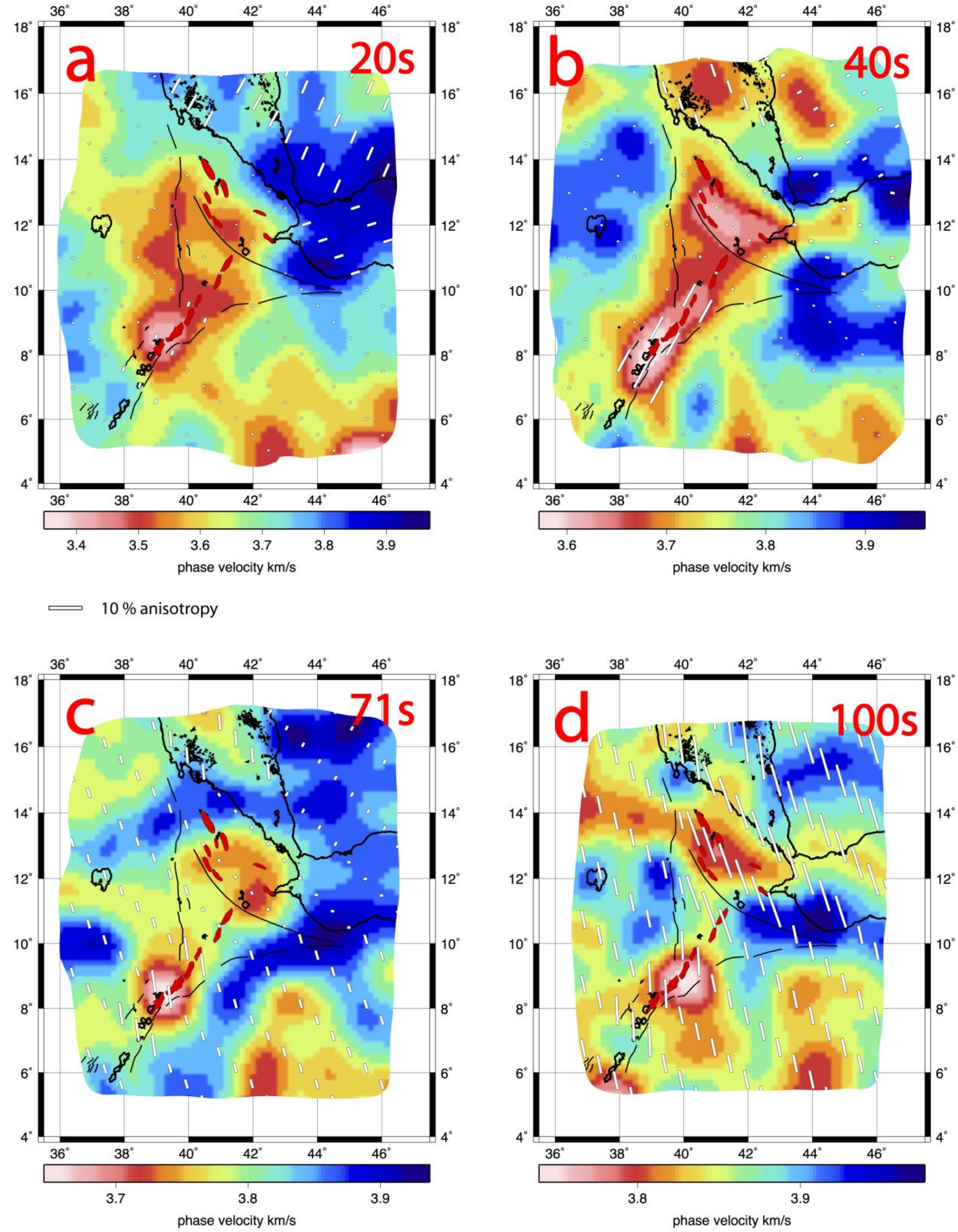

Supplementary Figure 6) Phase velocity maps. 2-dimensional phase velocity maps for each period superimposed with azimuthal anisotropy.

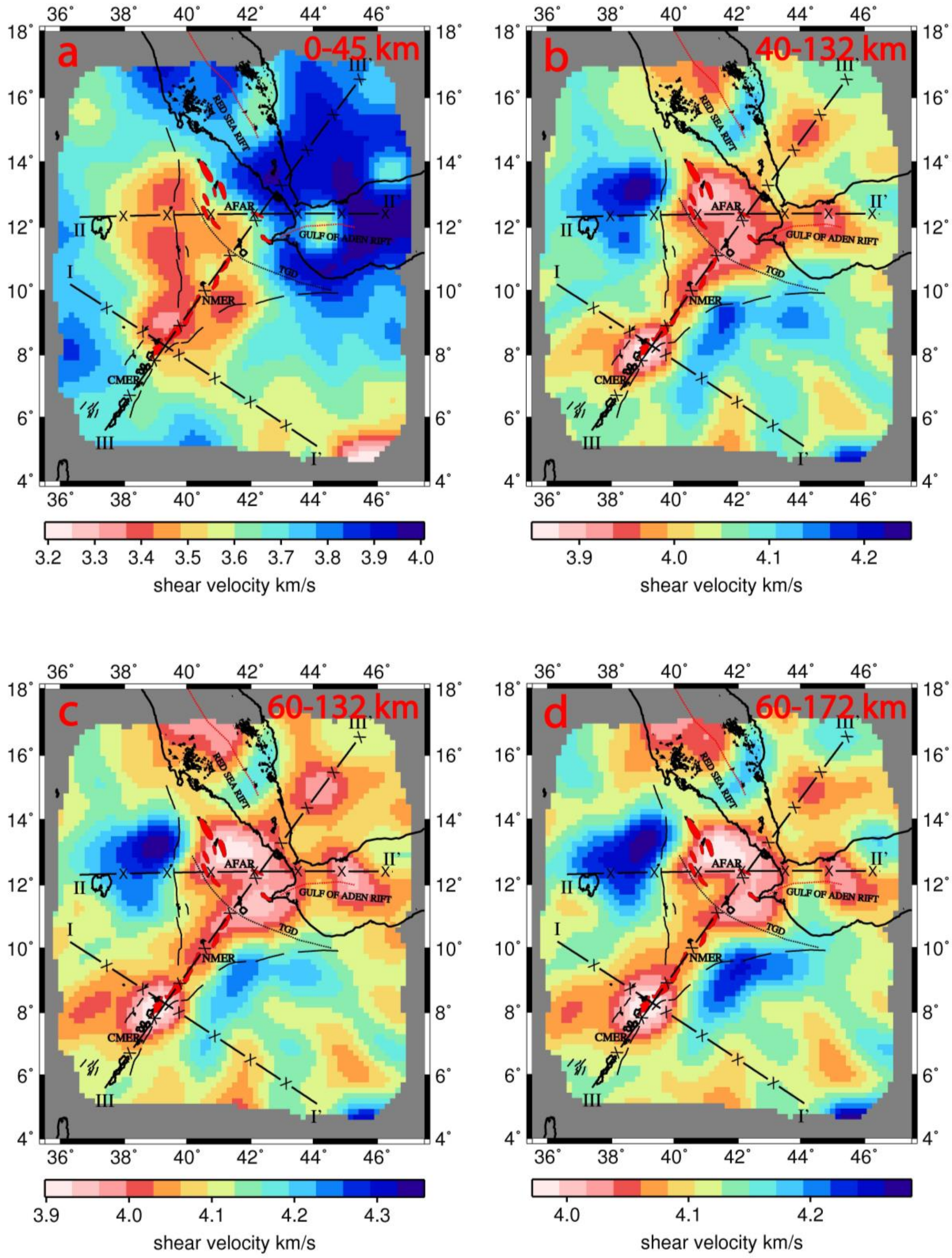

Supplementary Figure 7) Shear velocity maps. Depth averaged shear velocity maps; 0-45 km (**a**), 40-132 km (**b**), 60-132 km **(c)** & 60-172 km **(d)**.

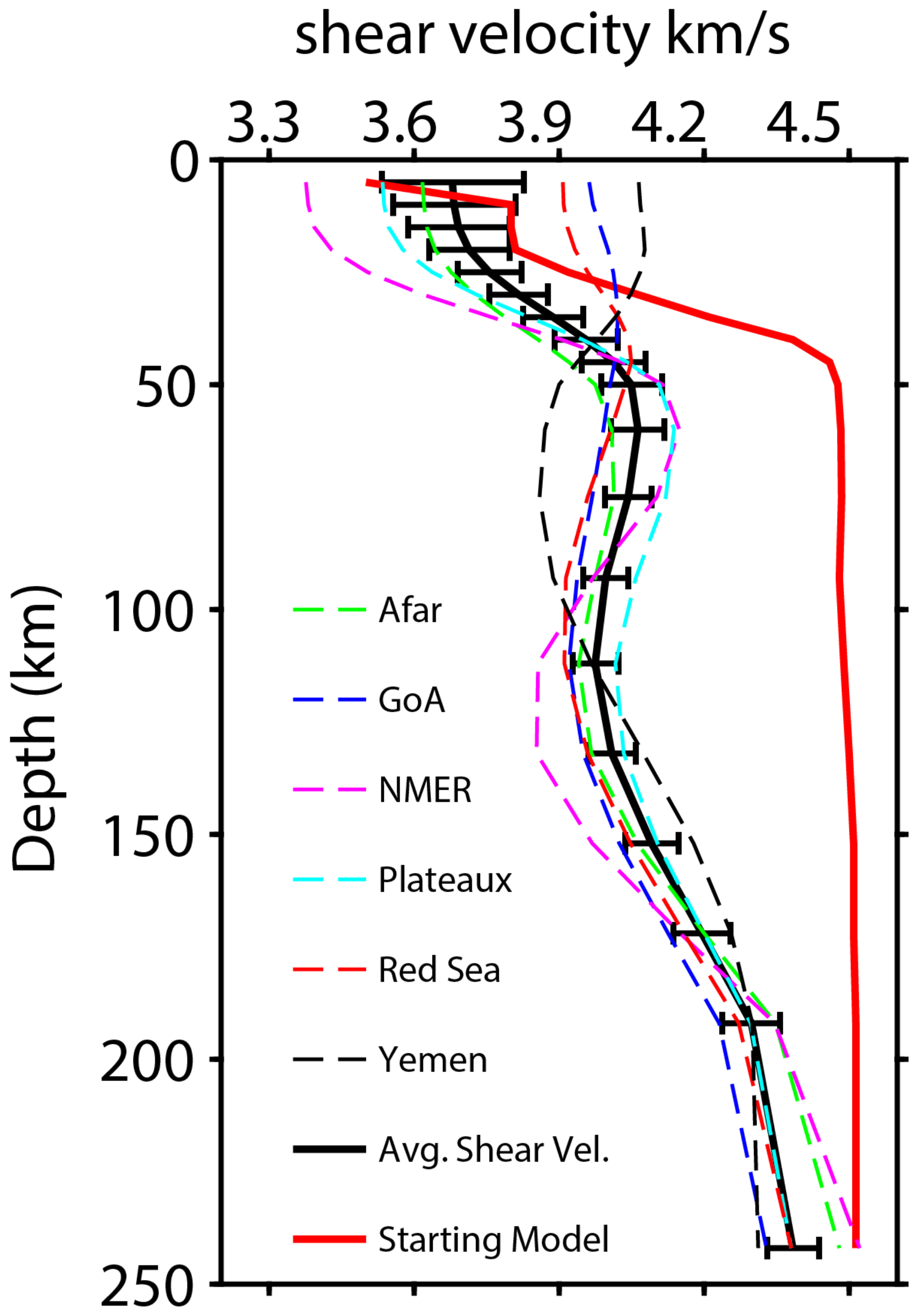

Supplementary Figure 8) 1-dimensional shear velocities. The average regional 1-dimensional shear velocity model (black) is up to 11% slower than the starting model (red), which combines CRUST 1.0 (0-40 km) and ak135 (40-250km), at depths >50 km. Errors are obtained from a Monte-Carlo estimate (100,000 random perturbations from our best fit model) showing the range of possible solutions to the inversion. Regional shear velocity profiles are shown as dashed lines.

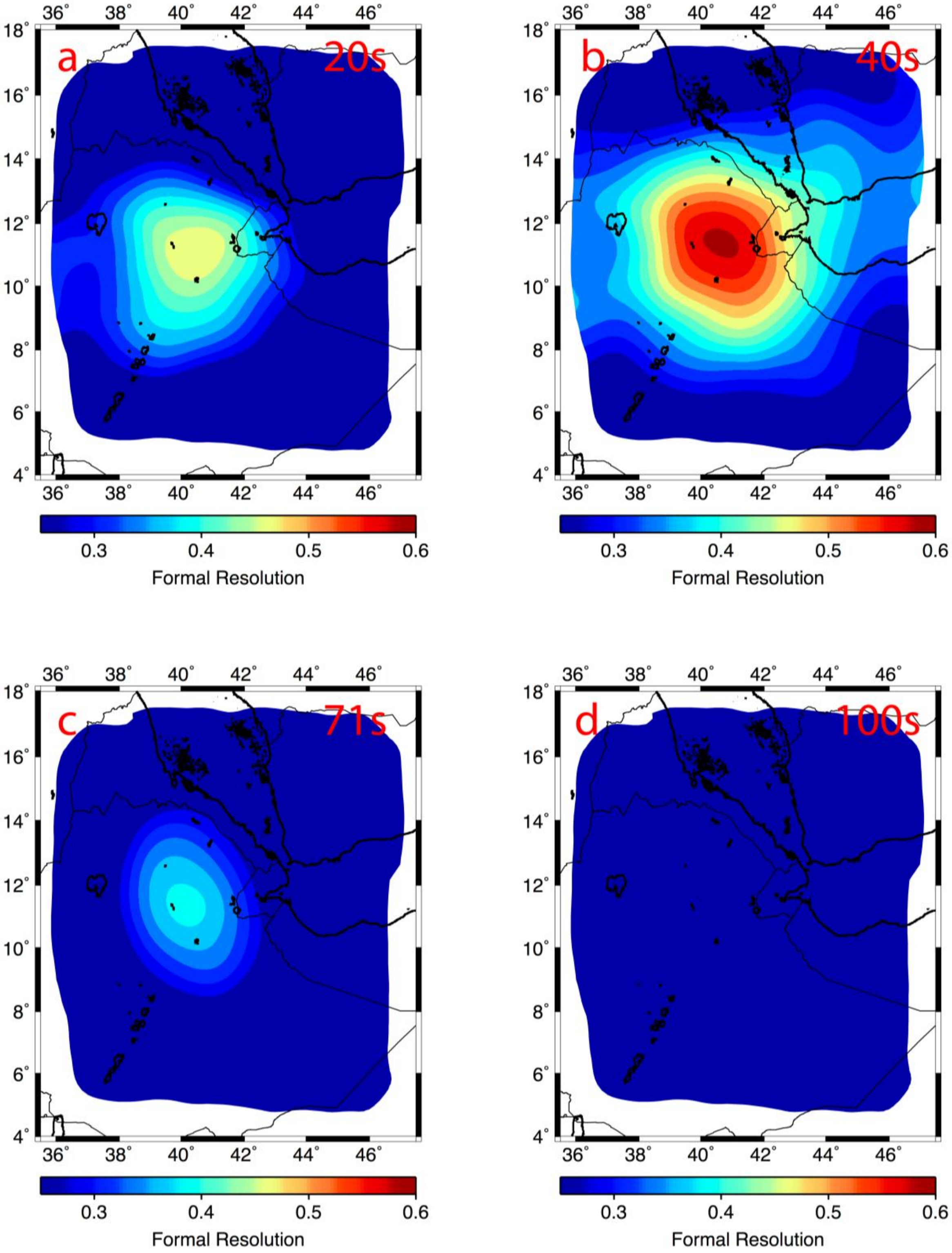

Supplementary Figure 9) Formal resolution. The node independence from formal resolution matrix for the 20 second period **(a),** 40 second period **(b),** 71 second period **(c)** and 100 second period **(d)**.

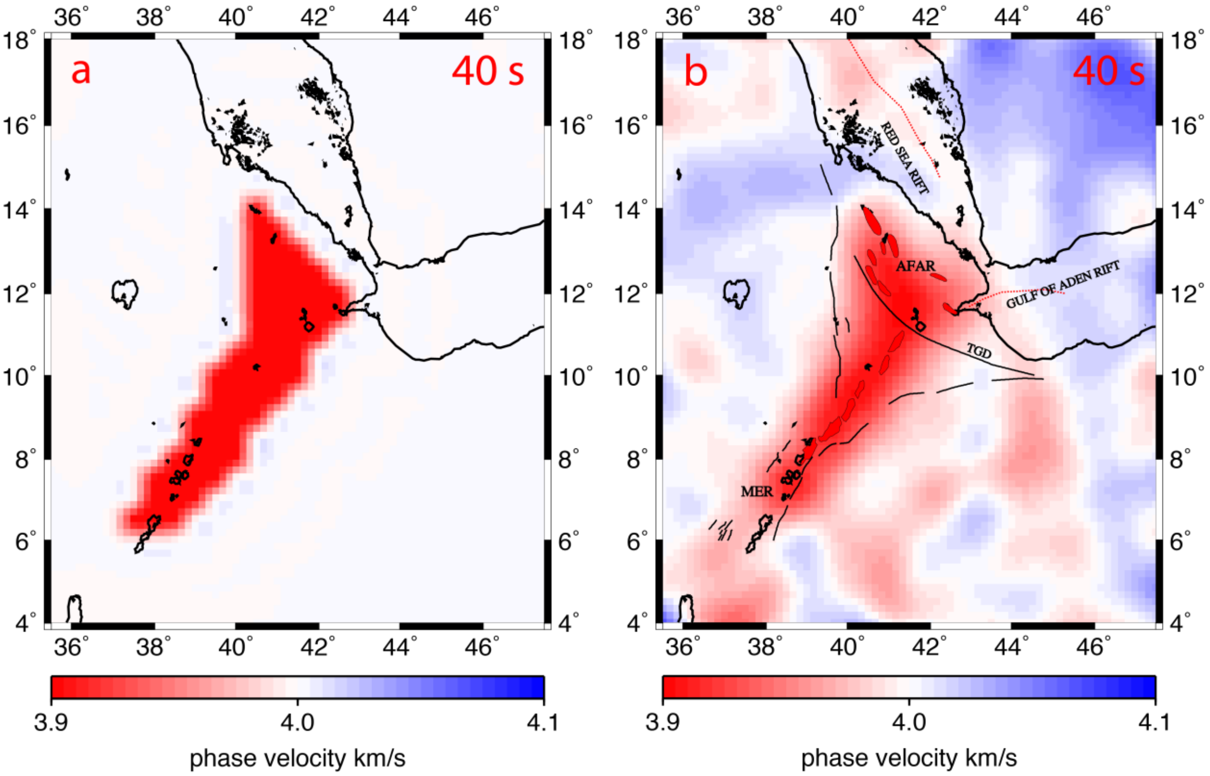

Supplementary Figure 10) Rift segmentation test. Synthetic 2-dimensional phase velocity map of low velocity within the rift at 40-seconds period **(a)**, recovered 2-dimensonal phase velocity map of low velocity with the rift at 40-seconds period **(b)**.

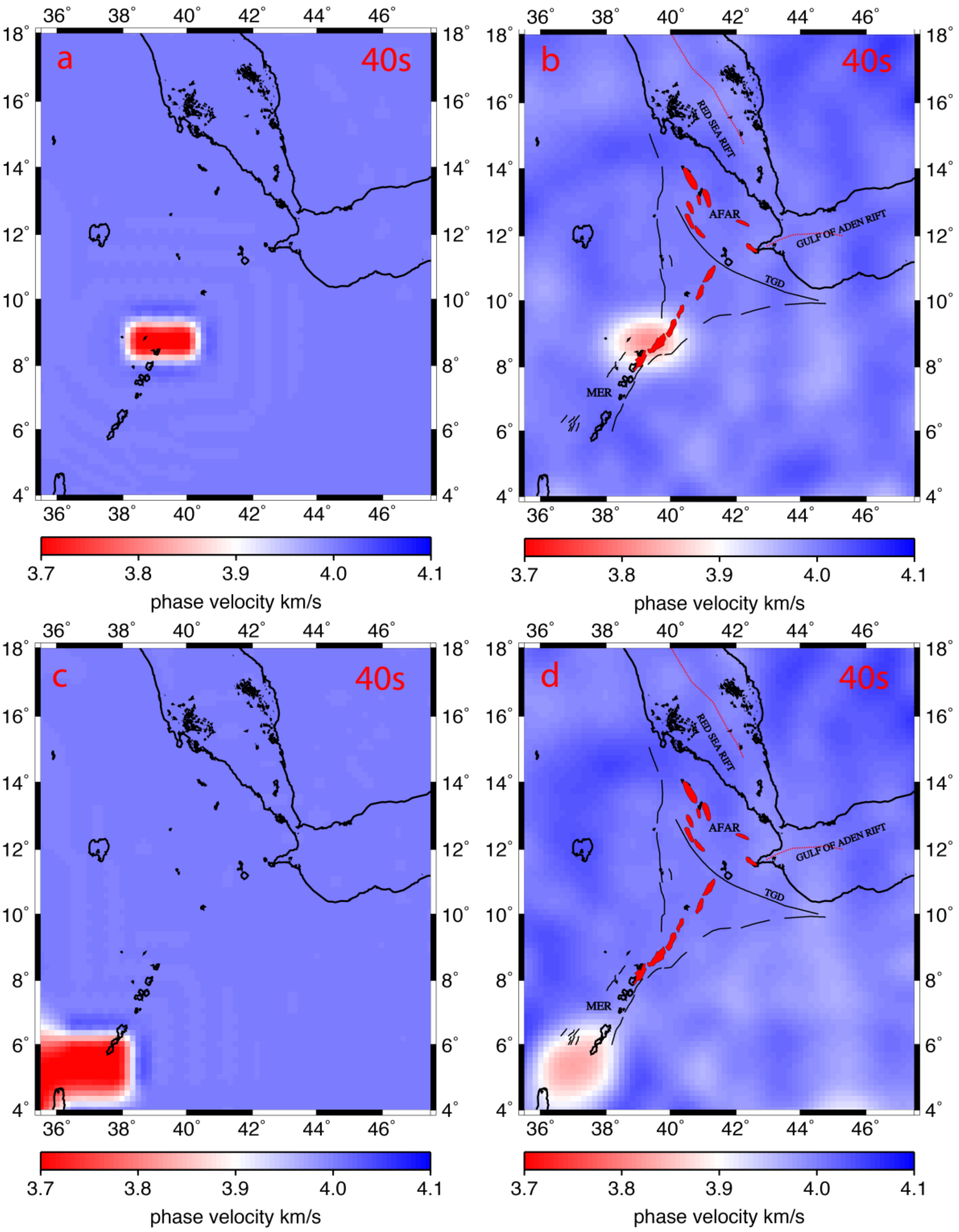

Supplementary Figure 11) Rift smearing test. Synthetic 2-dimensional phase velocity map of low velocity within the NMER at 40-seconds period **(a)**, recovered 2-dimensonal phase velocity map of low velocity within the NMER at 40-seconds period **(b)**, synthetic 2-dimensional phase velocity map of low velocity south of the NMER at 40-seconds period **(c)**,

recovered 2-dimensonal phase velocity map of low velocity south of the NMER at 40-seconds period **(d).**

Supplementary Tables

Depth (km)	Average Shear Velocity (km/s)	Shear Velocity Error (km/s)	Period (s)	Average Phase Velocity (km/s)	Phase Velocity Error x3 (km/s)
5	3.3976	0.1467	20	3.4371	0.0177
10	3.4004	0.1267	22	3.4874	0.0141
15	3.4051	0.1049	25	3.5401	0.0123
20	3.4238	0.0829	29	3.5917	0.0108
25	3.4702	0.0668	33	3.6300	0.0090
30	3.5470	0.0605	40	3.6677	0.0096
35	3.6492	0.0622	50	3.7052	0.0087
40	3.7650	0.0654	67	3.7606	0.0117
45	3.8798	0.0660	73	3.7843	0.0126
50	3.9804	0.0630	77	3.7904	0.0155
60	4.0584	0.0558	81	3.8097	0.0129
75	4.1000	0.0482	91	3.8456	0.0147
93	4.0989	0.0466	100	3.8692	0.0174
112	4.0915	0.0471	111	3.8929	0.0177
132	4.1130	0.0493	125	3.9708	0.0198
152	4.1680	0.0549			
172	4.2426	0.0597			
192	4.3231	0.0597			
242	4.4002	0.0536			

Supplementary Table 1) Average phase and shear velocity values. Values and errors for average 1-dimensional shear velocity profile and phase velocity dispersion curve.
